# Black Hole Entropy: A Closer Look

**DOI:** 10.3390/e22010017

**Published:** 2019-12-22

**Authors:** Constantino Tsallis

**Affiliations:** 1 Centro Brasileiro de Pesquisas Físicas and National Institute of Science and Technology of Complex Systems, Rua Xavier Sigaud 150, Rio de Janeiro 22290-180, RJ, Brazil; tsallis@cbpf.br; 2 Santa Fe Institute, 1399 Hyde Park Road, Santa Fe, NM 87501, USA; 3 Complexity Science Hub Vienna, Josefstädter Strasse 39, 1080 Vienna, Austria

**Keywords:** black holes, nonadditive entropies, thermodynamics, complex systems

## Abstract

In many papers in the literature, author(s) express their perplexity concerning the fact that the (3+1) black-hole ‘thermodynamical’ entropy appears to be proportional to its area and not to its volume, and would therefore seemingly be nonextensive, or, to be more precise, subextensive. To discuss this question on more clear terms, a non-Boltzmannian entropic functional noted $S_{\delta }$ was applied [Tsallis and Cirto, Eur. Phys. J. C 73, 2487 (2013)] to this complex system which exhibits the so-called area-law. However, some nontrivial physical points still remain open, which we revisit now. This discussion is also based on the fact that the well known Bekenstein-Hawking entropy can be expressed as being proportional to the event horizon area divided by the square of the Planck length.

## 1. Introduction

We frequently verify perplexity by various authors that the entropy of a black hole appears to be proportional to its area whereas it was expected to be proportional to its volume. Such perplexity is tacitly or explicitly based on a sort of belief (i) that a black hole is a three-dimensional system, and (ii) that its thermodynamic entropy is to be understood as its Boltzmann-Gibbs (BG) one. We critically focus here on both assumptions, which we argue to be by no means obvious.

Let us start by reminding some of those—by now classical—indications of a sort of surprise.

For instance, Hawking, in the Abstract of his 1976 paper [1], he states *A black hole of given mass, angular momentum, and charge can have a large number of different unobservable internal configurations which reflect the possible different initial configurations of the matter which collapsed to produce the hole. The logarithm of this number can be regarded as the entropy of the black hole and is a measure of the amount of information about the initial state which was lost in the formation of the black hole. [...] the entropy is finite and is equal to $c^{3}A/4G\hbar$, where A is the surface area of the event horizon or boundary of the black hole.*

Also, Maddox, in his 1993 letter [2], he writes *So why is the entropy of a black hole proportional to the square of its radius, and not to the cube of it? To its surface area rather to its volume?*

Das and Shankaranarayanan [3] specifically comment in 2006 that *The area (as opposed to volume) proportionality of BH entropy has been an intriguing issue for decades*.

Kolekar and Padmanabhan [4] argue in 2011 that *As the box is moved closer to the horizon with one (leading) edge of the box at about Planck length ($L_{P}$) away from the horizon, the entropy shows an area dependence rather than a volume dependence. [...] Thus the contribution to the entropy comes from only a fraction $O(L_{P}/K)$ of the matter degrees of freedom and the rest are suppressed when the box approaches the horizon. [...] Thus the extensive property of entropy no longer holds and one can check that it does not hold even in the weak field limit discussed above when L>>λ that is, when gravitational effects subdue the thermal effects along the direction of the gravitational field.*

Many other papers (see, for instance, [5,6,7,8,9,10,11,12,13,14,15,16,17,18,19,20,21,22,23,24,25]) do focus on such issues. In all of these, the entropy that is shown to be proportional to the area, instead of the volume, is the Boltzmann-Gibbs-von Neumann-Shannon one, from now on referred to as $S_{BG}$. For a discrete number *W* of microscopic states, $S_{BG}$ is defined as

(1) $$S_{BG}=-k\sum_{i=1}^{W}p_{i}\ln p_{i}\;\;\;\left(\;\sum_{i=1}^{W}p_{i}=1\right)\;,$$

with

(2) $$S_{BG}=k\ln W\;,$$

in the case of equal probabilities, *k* being the Boltzmann constant.

For quantum systems, we have

(3) $$S_{BG}=-kTr\rho \ln\rho \;\;\;\left(\;Tr\rho =1\right)\;,$$

ρ being the density matrix.

For classical systems, we have

(4) $$S_{BG}=-k\int dx\;p\left(x\right)\ln p\left(x\right)+constant\;\;\;\left(\;\int dx\;p(x)=1\right)\;.$$

So, in many of the above papers what is found for the black hole is that

(5) $$\frac{S_{BG}}{k}\propto \frac{A}{L_{P}^{2}}\;,$$

with the Planck length $L_{P}\equiv \sqrt{\frac{hG}{c^{3}}}$. In all such papers it is tacitly assumed that $S_{BG}$ is the thermodynamic entropy of the black hole. But, as argued in [24,26], there is no reason at all for this being a correct assumption, especially so given the fact that the thermodynamic entropic extensivity would then be violated.

More generally speaking, this brings us to the so called “area laws” [27] in quantum entangled systems. More precisely, if we have a *d*-dimensional system or subsystem with linear size *L*, its hyper-volume (or volume for short) is given by $L^{d}$, and its hyper-area (or area for short) is given by $L^{d-1}$. In such systems, it is currently obtained $\frac{S_{BG}}{k}\propto {\left[\frac{L}{L_{0}}\right]}^{d-1}$ (in the $L/L_{0}\to \infty$ limit), instead of ${\left[\frac{L}{L_{0}}\right]}^{d}$, as would in principle be thermodynamically expected, where $L_{0}$ is a microscopic reference length (for instance, for black holes, it is $L_{0}=L_{P}$). This anomaly is referred to in the literature as the area law. In the d=1 cases, it is currently obtained (see [28,29,30] and references therein) $\frac{S_{BG}}{k}\propto \ln\frac{L}{L_{0}}$, instead of $\frac{L}{L_{0}}$, as a priori expected from thermodynamics. Both d>1 and d=1 cases can be unified under the compact form

(6) $$\frac{S_{BG}}{k}\propto \frac{{\left[L/L_{0}\right]}^{d-1}-1}{d-1}\equiv \ln_{2-d}\left[L/L_{0}\right]\;,$$

with [31]

(7) $$\ln_{q}z\equiv \frac{z^{1-q}-1}{1-q}\;\;\;\left(z>0;\;q\in R\right)\;.$$

It is our standpoint that, whenever the *additive* [An entropic functional $S(\left\{p_{i}\right\})$ is said additive if $S\left(A+B\right)\equiv S(\left\{p_{i}^{A}\;p_{j}^{B}\right\}=S\left(\left\{p_{i}^{A}\right\}\right)+S\left(\left\{p_{j}^{B}\right\}\right)\equiv S\left(A\right)+S\left(B\right)$, for arbitrary $\left\{p_{i}^{A},p_{j}^{B}\right\}$. Consequently, $S_{BG}$ is additive whereas $S_{q}$ (for q≠1), $S_{\delta }$ (for δ≠1), and $S_{q,\delta }$ (for (q,δ)≠(1,1)) are nonadditive] entropic functional $S_{BG}$ is thermodynamically nonextensive, the entropic functional to be used for all thermodynamical issues is not given by Equations (1)–(4), but by a nonadditive one instead, such as [32,33]

(8) $$S_{q}=k\frac{1-\sum_{i=1}^{W}p_{i}^{q}}{q-1}=k\sum_{i=1}^{W}p_{i}\ln_{q}\frac{1}{p_{i}}\;\;\;\left(\;\sum_{i=1}^{W}p_{i}=1;\;q\in R;\;S_{1}=S_{BG}\right)\;,$$

with

(9) $$S_{q}=k\ln_{q}W\;,$$

in the case of equal probabilities, or such as [26]

(10) $$S_{\delta }=k\sum_{i=1}^{W}p_{i}{\left[\ln\frac{1}{p_{i}}\right]}^{\delta }\;\;\;\left(\;\sum_{i=1}^{W}p_{i}=1;\;\delta \in R;\;S_{1}=S_{BG}\right)\;,$$

with

(11) $$S_{\delta }=k{\left[\ln W\right]}^{\delta }\;,$$

in the case of equal probabilities, or even such as [24]

(12) $$S_{q,\delta }=k\sum_{i=1}^{W}p_{i}{\left[\ln_{q}\frac{1}{p_{i}}\right]}^{\delta }\;\;\;\left(\;\sum_{i=1}^{W}p_{i}=1;\;\left(q,\delta \right)\in R^{2};\;S_{q,1}=S_{q};\;S_{1,\delta }=S_{\delta };\;S_{1,1}=S_{BG}\right)\;,$$

with

(13) $$S_{q,\delta }=k{\left[\ln_{q}W\right]}^{\delta }\;,$$

in the case of equal probabilities. Many other nonadditive entropies are available in the literature, for instance [34,35,36,37,38,39,40,41].

The outstanding role we attribute to the thermodynamical extensivity of the entropy, in contrast to the additivity or nonadditivity of the specific entropic functional that we are adopting, comes from (i) the lack (as far as we are aware today) of physical necessity of violating the Legendre structure of thermodynamics (see [24,26] and references therein), and (ii) the fact that, for both simple and complex probabilistic systems [42,43], the central probability currently defined in the Large Deviation Theory appears to vanish, for infinitely large systems, as a simple function (exponential or some natural generalization of it) of (the so-called rate function) × (a relative entropy per particle) ×N, where *N* characterizes the size of the system. Notice that (i) and (ii) are perfectly consistent to each other since both yield the extensivity of the total entropy of the system.

Before proceeding on let us illustrate, with two simple equal-probability examples, how nonadditive entropies can solve the lack of thermodynamical extensivity of the BG entropy which frequently occurs in complex systems. The first of them, introduced in [44] and further explored in [45], focuses on the very frequent class of systems whose total number *W* of microscopic states scales like $W\left(N\right)∼N^{\rho }\;\left(\rho >0\right)$, where *N* denotes the number of particles or of degrees of freedom of the system. The use of the BG entropy leads to $S_{BG}\left(N\right)=k\ln W\left(N\right)\propto \ln N$, which is thermodynamically nonextensive (subextensive in fact) and therefore inadmissible. If we use instead $S_{q}\left(N\right)=k\ln_{q}W\left(N\right)\propto N$ if q=1−1/ρ, therefore $S_{1-1/\rho }$ is thermodynamically extensive hence admissible. The second example, introduced in [26] and further explored in [24], focuses on $W\left(N\right)∼\nu^{N^{\gamma }}\;\left(\nu >1;\;0<\gamma <1\right)$. The use of the BG entropy leads to $S_{BG}\left(N\right)=k\ln W\left(N\right)\propto N^{\gamma }$, which is thermodynamically nonextensive (subextensive in fact) and therefore inadmissible. If we use instead $S_{\delta }\left(N\right)=k{\left[\ln W\left(N\right)\right]}^{\delta }\propto N$ if δ=1/γ, therefore $S_{\delta =1/\gamma }$ is thermodynamically extensive hence admissible. What these two examples have in common is that, since W(N) scales, due to relevant correlations, differently from the usual simple case (namely $W\left(N\right)\propto \mu^{N}\;\left(\mu >1\right)$), the use of the additive functional $S_{BG}$ violates thermodynamics, whereas the use of appropriate nonadditive entropic functionals (such as $S_{q}$, $S_{\delta }$ and others) satisfies thermodynamics. This perspective beautifully reinforces Einstein 1949 statement [46]. Sacrificing the entropic additivity of $S_{BG}$ is a small price to pay in order to satisfy thermodynamics and its Legendre-transform structure, very similarly to sacrificing Galileo’s additivity of one-dimensional composition of velocities $v_{12}=v_{1}+v_{2}$ (and replacing it by Einstein’ s nonadditive composition $v_{12}=\frac{v_{1}+v_{2}}{1+v_{1}v_{2}/c^{2}}$) is a small price to pay in order to satisfy Lorentz invariance and therefore magnificently unify mechanics and Maxwell electromagnetism.

## 2. Dimensionality of the System

The spatial dimensionality of a system might be non unique. For example, a chocolate Easter Egg is embedded in a three-dimensional space, but if the width of the chocolate is fixed to say 3 mm for eggs of various sizes, the system essentially scales as a two-dimensional one in what concerns the total amount of chocolate. In what concerns thermodynamics, the spatial dimensionality of a (3+1) black hole depends on whether its bulk (inside its event horizon or boundary) has or not non negligible amount of matter oranalogous physical information. If that matter or information is non negligible, the thermodynamical entropy of the black hole must scale as $L^{d}$ with d=3, where *L* stands for its linear size. If that matter or information is negligible, the thermodynamic entropy of the black hole must scale as $L^{d}$ with d=2. Once, and only once, the amount of mass (or equivalent information) in the bulk of the black hole has been neatly established, we can talk about its entropy, expected to scale as $L^{d}$ in all cases. This is why, in [24], we distinguish (in Italic) whether the entropic functional $S_{BG}$ is thermodynamically admissible or not. Indeed, the Bekenstein-Hawking entropy, which scales as the area of the black hole, certainly is thermodynamically admissible (i.e., extensive) if that black hole is to be considered as an essentially two-dimensional object. But it becomes non admissible if it is considered as an essentially three-dimensional object. In that case, appropriate nonadditive entropic functionals are expected to play a most relevant role. Indeed, appropriate values of the indices (say *q*, δ) of those nonadditive entropies can restore the obligatory thermodynamical extensivity (see further details in [24,26] for why the thermodynamical entropy is obligatorily extensive, i.e., proportional to $L^{d}$).

## 3. Subtlety about the Entropic Extensivity of Nonadditive Entropic Functionals

For quantum d=1 *L*-sized subsystems such as those focused on in [28], it is known that $S_{BG}\propto \ln L$, which exhibits the thermodynamical inadmissibility of $S_{BG}$. It is therein shown that, instead, $S_{q}\propto L$ if

(14) $$q=\frac{\sqrt{9+{\left(\bar{c}\right)}^{2}}-3}{\bar{c}}\;,$$

where $\bar{c}$ denotes the central charge of the system. In other words, this is the thermodynamically correct entropy for the subsystem, with which other thermodynamical properties ought to be calculated.

Let us, however, comment at this point on a possible error that can easily emerge. It is known, for the above subsystems, that

(15) $$\frac{S_{BG}\left(L\right)}{k}=\frac{\bar{c}}{3}\ln L+\ln b+\ldots =ln\left[bL^{\bar{c}/3}\right]+\ldots \;\;\left(b>1\right)$$

But if we (wrongly) assume equal probabilities, we will (wrongly) use the equal probability expression $S_{BG}=k\ln W$ , thus identifying $W∼bL^{\bar{c}/3}$. This corresponds to the power-law class $W\propto L^{\rho }\;\;\left(\rho >0;\;L\to \infty \right)$, whose extensive entropy is the nonadditive one $S_{q}$ with $q=1-1/\rho =1-3/\bar{c}$ [44,45]. This expression is in variance with Equation (14) and is therefore definitively wrong for all $\bar{c}<\infty$ ! In this illustration, the error does not affect the class of nonadditive entropic functionals $S_{q}$, but it concerns the correct value of the index *q*. Physically speaking, this confusion arises from the fact that relation (15) is valid for the subsystem; let us recall that the kinetic temperature of the total system vanishes, whereas the effective temperature of a *L*-sized subsystem is finite [47]. This is in strong variance with infinity, which would correspond to the micro-canonical ensemble and would therefore justify the use of the equal-probability hypothesis.

In [24] we have used, without further justification, the equal-probability hypothesis in order to determine the value of the index δ of the class of nonadditive entropic functionals $S_{\delta }$. This working assumption for black holes was done to fix a possible value δ=d/(d−1)>1 upon which further considerations could be developed, to show in particular that the class $S_{\delta }$ constitutes a plausible one to restore the desired extensivity for the thermodynamical entropy. However, this specific value of δ (and of the class $S_{\delta }$) requires further analysis. At the present stage we can not categorically exclude that the correct class could be say $S_{q}$, or even $S_{q,\delta }$, with indices (q,δ) to be determined on a fully justified basis. This certainly constitutes a challenging program.

## 4. Final Remarks

The Bekenstein-Hawking entropy $S_{BH}$ (BH stands for Bekenstein-Hawking) for a black hole was established as

(16) $$S_{BH}=\frac{k}{4}\frac{A_{H}}{G\hbar /c^{3}}\;,$$

where $A_{H}$ is the event horizon area. This expression is thermodynamically admissible only if the black hole is to be considered a d=2 object, but it is not admissible if it is assumed to be a d=3 one. The alternative entropy suggested in [24] under the assumption that the black hole is a d=3 object is thermodynamically admissible and it comes out to be given by

(17) $$\frac{S_{\delta =3/2}}{k}\propto {\left[\frac{S_{BH}}{k}\right]}^{3/2}\;.$$

This expression yields

(18) $$\frac{S_{\delta =3/2}}{k}\propto {\left[\frac{L_{H}}{L_{P}}\right]}^{3}\;,$$

where $L_{H}=\sqrt{A_{H}}$ and the proportionality factors are pure numbers. This expression is clearly plausible since it implies that the black hole (if and only if it is assumed to be three-dimensional) thermodynamical entropy is proportional to its volume divided by the microscopic volume $L_{P}^{3}$. Let us also remark that, like $S_{BH}$, this extensive entropy $S_{\delta =3/2}$ is given in terms of all four fundamental physical constants (G,1/c,1/k,h) [26,48], where (0,0,0,0) corresponds to plain Newtonian mechanics (by plain, we refer to the fact that no particular force is assumed, such as say the gravitational one, here characterized by *G*). The fact that 1/k=0 is compatible with classical mechanics deserves a comment. Diverging *k* is basically equivalent to diverging temperature *T* (since they always appear in the form kT), i.e., it corresponds to the micro-canonical ensemble, which is ultimately characterized by the size of the phase space compatible with a given total energy. This phase space is determined exclusively by the dynamics, say Newtonian one. This is also related to the fact that, for probabilistically independent systems *A* and *B*, we have, for say the entropy $S_{q}$, that $\frac{S_{q}\left(A+B\right)}{k}=\frac{S_{q}\left(A\right)}{k}+\frac{S_{q}\left(B\right)}{k}+\left(1-q\right)\frac{S_{q}\left(A\right)}{k}\frac{S_{q}\left(B\right)}{k}$, hence $S_{q}\left(A+B\right)=S_{q}\left(A\right)+S_{q}\left(B\right)+\frac{1-q}{k}S_{q}\left(A\right)S_{q}\left(B\right)$. Consequently, in the limit 1/k=0 we recover the BG additivity, namely $S_{BG}\left(A+B\right)=S_{BG}\left(A\right)+S_{BG}\left(B\right)$. This is consistent with the fact that, at T→∞, all statistics (Fermi-Dirac, Bose-Einstein, Gentile parastatistics, *q*-statistics) asymptotically merge into one and the same behaviour, namely that of the Maxwell-Boltzmann microcanonical ensemble.

Nowadays many studies (see, for instance, [49,50,51,52,53,54,55,56,57,58,59,60]) address entropic issues based on nonadditive entropic functionals such as $S_{q}$, $S_{\delta }$ or $S_{q,\delta }$ (see also [61], where possible alternative interpretations of the entropic forms have been advanced). They focus on black holes, cosmological issues (e.g., dark energy), and various kinds of quantum-entangled systems. These and similar explorations certainly are very welcome since they might eventually clarify the whole discussion. 
